# Social Determinants of Cardiovascular Disease

**DOI:** 10.1161/CIRCRESAHA.121.319811

**Published:** 2022-03-04

**Authors:** Tiffany M. Powell-Wiley, Yvonne Baumer, Foster Osei Baah, Andrew S. Baez, Nicole Farmer, Christa T. Mahlobo, Mario A. Pita, Kameswari A. Potharaju, Kosuke Tamura, Gwenyth R. Wallen

**Affiliations:** Social Determinants of Obesity and Cardiovascular Risk Laboratory, National Heart, Lung, and Blood Institute (T.M.P.-W., Y.B., F.O.B., A.S.B., C.T.M., M.A.P., K.A.P.), National Institutes of Health, Bethesda, MD.; Intramural Research Program, National Institute on Minority Health and Health Disparities (T.M.P.-W.), National Institutes of Health, Bethesda, MD.; Neighborhood Social and Geospatial Determinants of Health Disparities Laboratory, Population and Community Sciences Branch, Intramural Research Program, National Institute on Minority Health and Health Disparities (K.T.), National Institutes of Health, Bethesda, MD.; Translational Biobehavioral and Health Disparities Branch, National Institutes of Health, Clinical Center, Bethesda, MD (N.F., G.R.W.).; The Pennsylvania State University (C.T.M.).

**Keywords:** cardiovascular diseases, health status disparities, social determinants of health, social justice, socioeconomic factors

## Abstract

Social determinants of health (SDoH), which encompass the economic, social, environmental, and psychosocial factors that influence health, play a significant role in the development of cardiovascular disease (CVD) risk factors as well as CVD morbidity and mortality. The COVID-19 pandemic and the current social justice movement sparked by the death of George Floyd have laid bare long-existing health inequities in our society driven by SDoH. Despite a recent focus on these structural drivers of health disparities, the impact of SDoH on cardiovascular health and CVD outcomes remains understudied and incompletely understood. To further investigate the mechanisms connecting SDoH and CVD, and ultimately design targeted and effective interventions, it is important to foster interdisciplinary efforts that incorporate translational, epidemiological, and clinical research in examining SDoH-CVD relationships. This review aims to facilitate research coordination and intervention development by providing an evidence-based framework for SDoH rooted in the lived experiences of marginalized populations. Our framework highlights critical structural/socioeconomic, environmental, and psychosocial factors most strongly associated with CVD and explores several of the underlying biologic mechanisms connecting SDoH to CVD pathogenesis, including excess stress hormones, inflammation, immune cell function, and cellular aging. We present landmark studies and recent findings about SDoH in our framework, with careful consideration of the constructs and measures utilized. Finally, we provide a roadmap for future SDoH research focused on individual, clinical, and policy approaches directed towards developing multilevel community-engaged interventions to promote cardiovascular health.

Social determinants of health (SDoH) are the economic, social, environmental, and psychosocial factors that influence health. Many have clear and significant impacts on cardiovascular health and disease (CVD) outcomes for populations globally.^1^ In the United States, cardiometabolic diseases caused more than an estimated 4.8 million deaths among working-age adults from 1990 to 2017.^2^ Moreover, the average working-age, all-cause mortality rates in the United States increased after 2010, in part, due to cardiometabolic diseases.^3^ These trends have been fueled by both rising obesity prevalence and large, widening health disparities based on social and environmental conditions that serve as SDoH.^2–5^

Recent events such as the death of George Floyd, the subsequent protests that ensued, and significant health disparities highlighted by the COVID-19 pandemic have all underscored the role of SDoH in CVD outcomes. Specifically, racial and ethnic minorities have had the highest mortality rates in the pandemic, in particular before the availability of vaccines, due to structural racism and the disproportionate health effects of discriminatory policies and disparities in employment, housing, education, and health care access.^6–8^ These events highlight an urgent need to better operationalize structural SDoH components within both clinical and policy interventions for CVD risk reduction to reduce health disparities.

There has also been recent interest in the mechanisms by which SDoH influence biologic pathways involved in CVD development.^9,10^ Described as the biology of adversity, these biologic sequelae of structural inequalities, long-standing discrimination, and adverse social and environmental conditions require targeted translational and basic research to (1) identify key biologic markers associated with SDoH that may serve as effective CVD risk prediction tools and (2) develop targets for tailored and personalized interventions for those at highest risk for poor CVD outcomes.^9^ In this review, we provide a framework for the role of SDoH in CVD development and summarize biologic mechanisms that may associate these SDoH to CVD pathogenesis. Furthermore, we examine recent studies focused on the integration of SDoH into clinical care and cardiovascular health interventions.

## A Health Equity-Focused Social Determinants of Health Framework

The lack of clarity on the mechanisms by which SDoH affect cardiovascular outcomes may be partially due to a limited focus on the lived experiences of vulnerable populations within existing SDoH frameworks.^11^ For instance, both the Centers for Disease Control and Prevention and the World Health Organization define SDoH as the conditions in the environments where people are born, live, learn, work, play, worship, and age that affect a wide range of health, functioning, and quality of life outcomes and risks.^12,13^ While extensive, both frameworks fail to highlight key social processes, such as stigmatization, discrimination, and marginalization, that facilitate social exclusion and the isolation of vulnerable populations (eg, racial and ethnic minorities, women, the elderly, the chronically ill, individuals with disabilities, lesbian, gay, bisexual, transgender, and queer (or questioning) individuals and others communities, and persons with low socioeconomic status [SES]).^14^ We argue that to work towards health equity, a SDoH framework should more critically focus on those most impacted by disparities given the disproportionate cardiovascular health effects of SDoH on these at-risk populations.

**Figure 1.**
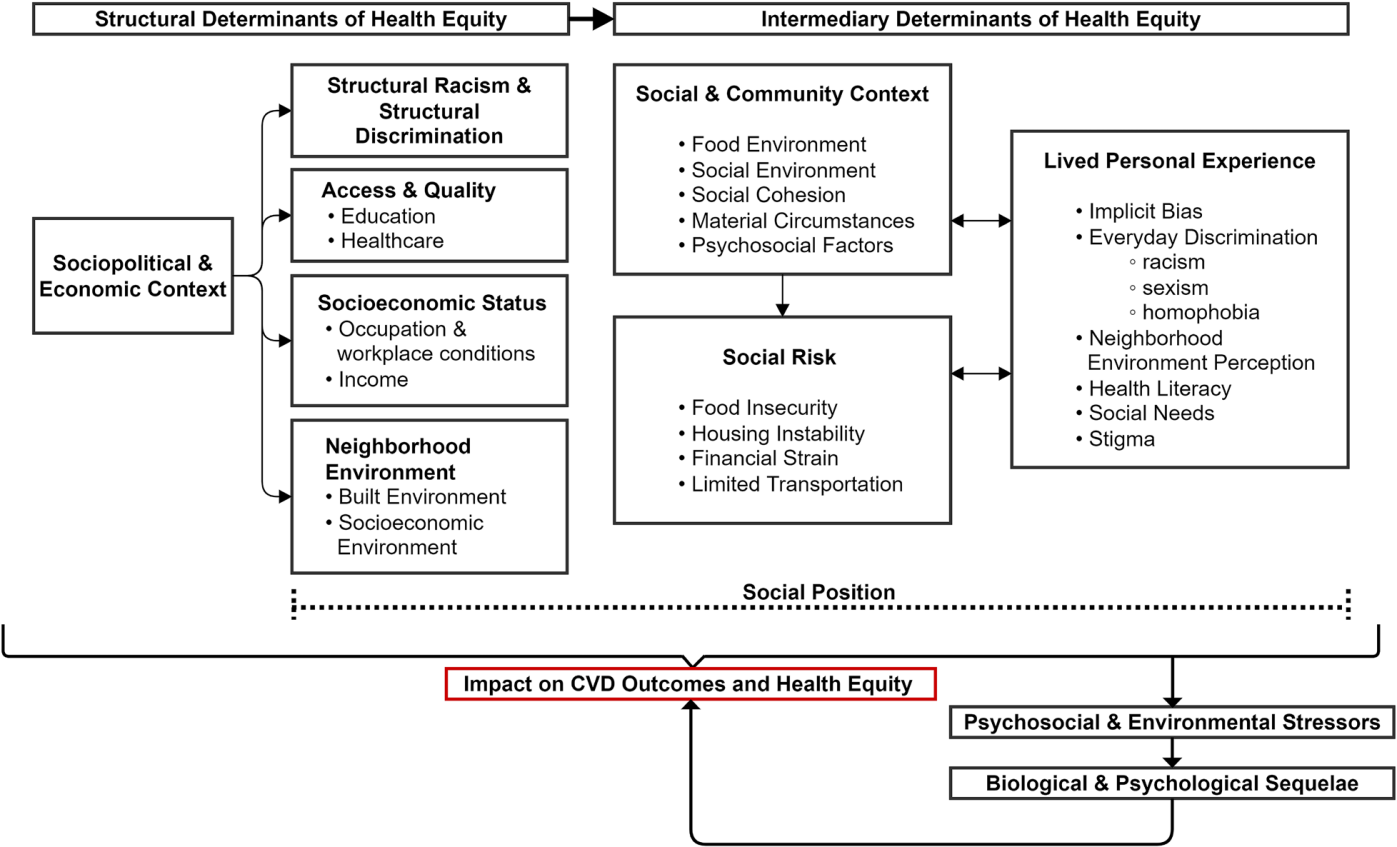
**A critical framework of social determinants of health.** The trickle-down effects of the sociopolitical and economic context shape social position and subsequent lived experiences of marginalized groups through the application of laws and policies within the social and community context. The everyday experience of othering such as discrimination, implicit bias, and stigma stems from structural determinants that shape social risk and an individual’s perception. The chronic effects of these experiences influence human biology and subsequent cardiovascular disease (CVD) outcomes through psychosocial and environmental stressors.

Therefore, our health equity-focused framework (Figure 1), revised from Baah et al,^14^ emphasizes the social position of vulnerable groups to highlight their lived experiences and perceptions that develop upon interaction with social and built environments. Structural and intermediary determinants constitute the 2 domains of the framework. Within the structural domain, sociopolitical and economic context (eg, governmental laws and economic policies) define access to, and the quality of health care and education, SES, or neighborhood environment, as well as exposure to structural racism and discrimination. These high-level health equity determinants influence the intermediary determinants, including social and community context (ie, food environment, social environment, and psychosocial factors), which ultimately define social risk through housing instability, food insecurity, financial strain, or limited transportation.^15^ This framework disentangles structural health equity determinants into key constructs which allows a focus on their roles in the lived personal experiences of vulnerable populations, which may include implicit bias, everyday discrimination or stigma. This framework also allows for the exploration of the intersectional effects of broader sociopolitical, cultural and economic factors and intermediary health determinants on social position. Eventually, adverse SDoH in this framework can chronically stress the biology of disadvantaged groups, impact cardiovascular health factors, and promote disparities in CVD outcomes.

**Figure 2.**
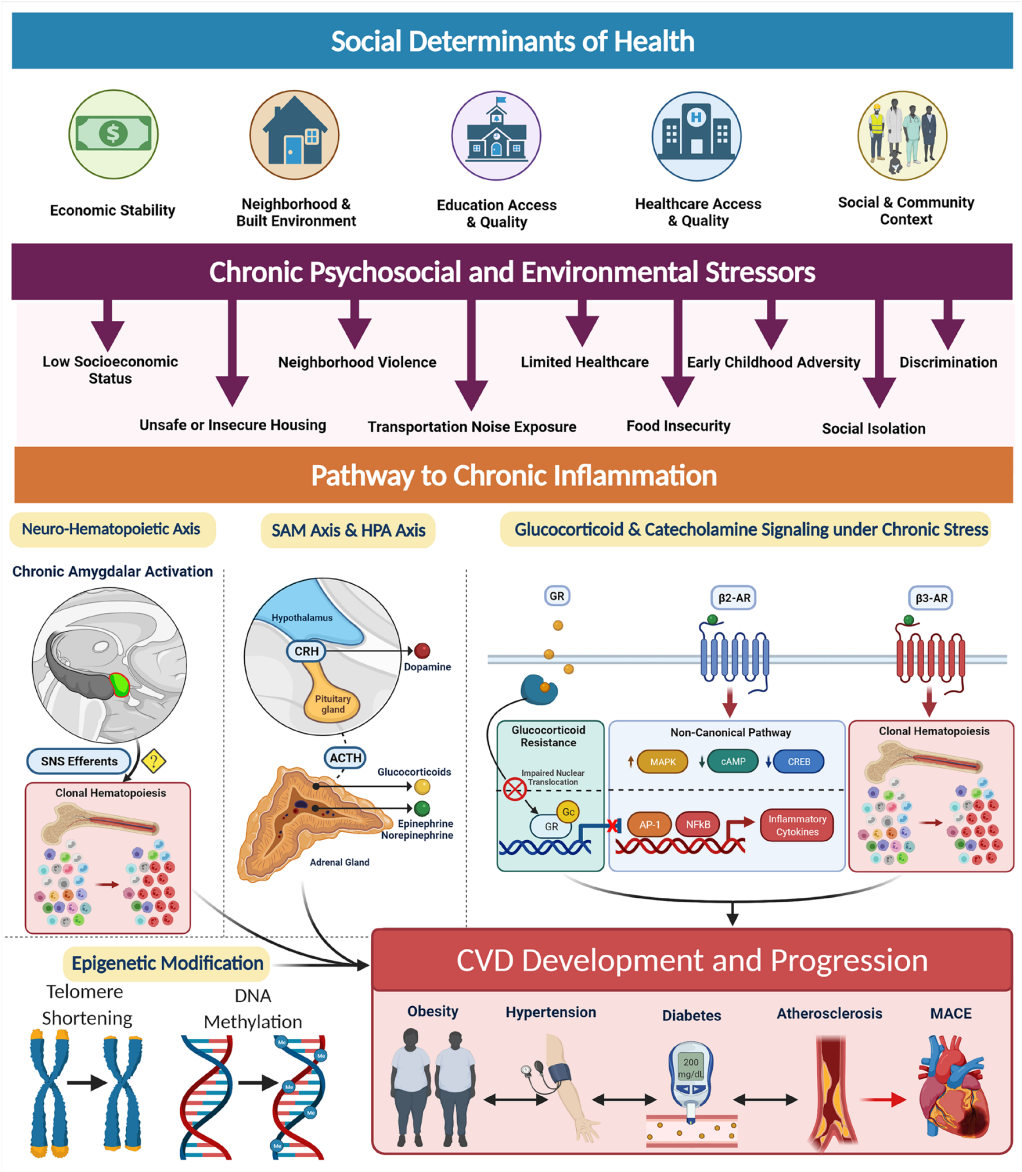
**The social determinants of health and the biology of adversity.** Social determinants of health encompass an individual’s economic stability, neighborhood and built environment, education access, health care access and their social and community relationships. These areas can be sources of chronic psychosocial stressors to individuals that suffer from low socioeconomic status, unsafe housing, neighborhood violence, limited access to health care, early childhood adversity, discrimination, increased noised exposure, food insecurity, and decreased sleep quality among others. Pathway to chronic inflammation: Biologic consequence of adversity promote pathways to chronic inflammation. Sympatho-adrenomedullary (SAM) axis and hypothalamic-pituitary-adrenal (HPA) axis: the SAM axis and the HPA axis are activated by psychosocial stress and regulate the production of catecholamines (dopamine, norepinephrine, and epinephrine) and glucocorticoids, respectively. Glucocorticoid and catecholamine signaling under chronic stress: (1) Glucocorticoid receptor (GR) shows impaired nuclear translocation and decreased anti-inflammatory gene transcription in chronic stress. (2) β-Adrenergic receptors (ARs) have been found to alter their gene signaling to a noncanonical pathway (via β-arrestin 2 scaffolding) that increases production of inflammatory cytokines which also upregulate NLRP3 (NLR family pyrin domain-containing 3) inflammasome activity. β3 Receptors have also been found to play a role in clonal hematopoiesis which may contribute to atherosclerotic plaque formation. Neurohematopoietic axis: Chronic amygdalar activation has been linked to clonal hematopoiesis, possibly by direct sympathetic nervous system (SNS) innervation of the bone marrow; stress-induced leukopoiesis has been directly linked to atherosclerotic plaques. All of these inflammatory processes lead to increased cardiovascular disease (CVD) risk factors, such as obesity, hypertension, diabetes, and atherosclerosis, ultimately contributing to major adverse cardiac events (MACE) and CVD mortality. ACTH indicates adrenocorticotropic hormone; AP-1, activating protein-1; CREB, cAMP response element-binding protein; CRH, corticotropin-releasing hormone; MAPK, mitogen-activated protein kinases; NF-κB, nuclear factor κ-light-chain-enhancer of activated B cells; and SNS, sympathetic nervous system. [Created with BioRender.com.]

**Figure 3.**
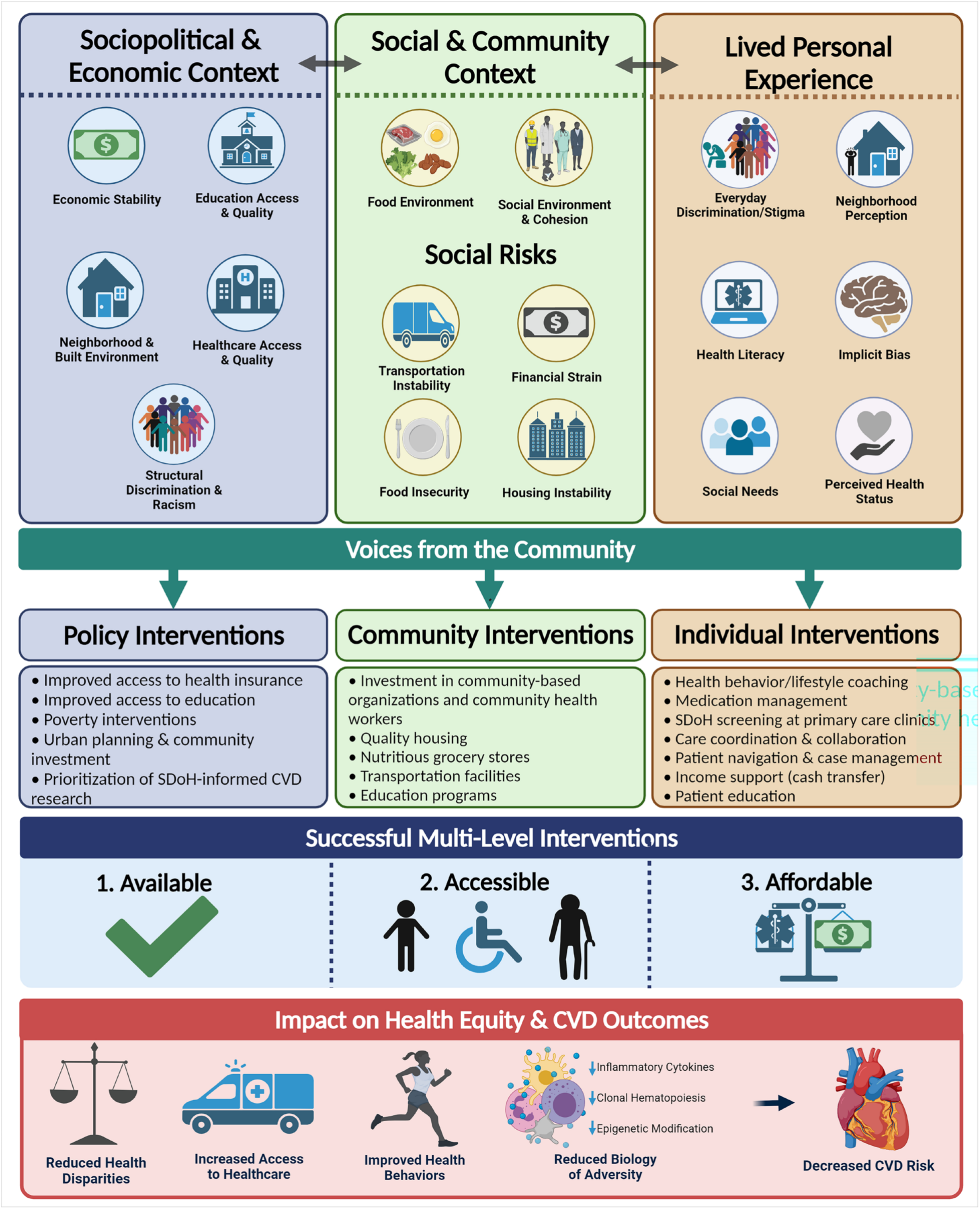
**Integration of the social determinants of health (SDoH) into multilevel cardiovascular health interventions.** Based on the previously presented critical framework of the social determinants of health, multiple levels of interventions at the policy, community, and individual levels are needed to address the sociopolitical, community, and lived experience contexts of cardiovascular health. Community input and engagement at all stages is necessary to develop well-informed interventions that provide available, accessible, and affordable resources to vulnerable populations. Ultimately, these successful multilevel interventions have direct impacts on cardiovascular disease (CVD) outcomes and health equity, such as reducing health disparities, improving health behaviors and access, and reducing the biologic impact of adverse conditions. [Created with BioRender.com.]

The constructs from the framework highlighted in this review were chosen based on evidence from longitudinal studies linking each to cardiovascular risk factors and CVD, as described in the following sections and outlined in Tables 1 and 2. We focus on these constructs to help harmonize evidence-based SDoH measures used in future research, especially in clinical trials where demographic variables, particularly race and ethnicity, are often used as a proxy for SDoH constructs.^16^ Understanding these key constructs is also critical to address the knowledge gaps in how SDoH constructs with limited or no available longitudinal data impact cardiovascular health of marginalized groups. While Figure 1 describes the health equity-focused social determinants of health framework, Figure 2 shows the biology of adversity, or the connections between SDoH and known biologic pathways which lead to chronic inflammation and CVD. Finally, we discuss the role and development of interventions at the individual, community, and policy levels that may improve SDoH, reducing the biologic effects of adversity and, ultimately CVD risk (Figure 3 and Table 3).

**Table 1.**
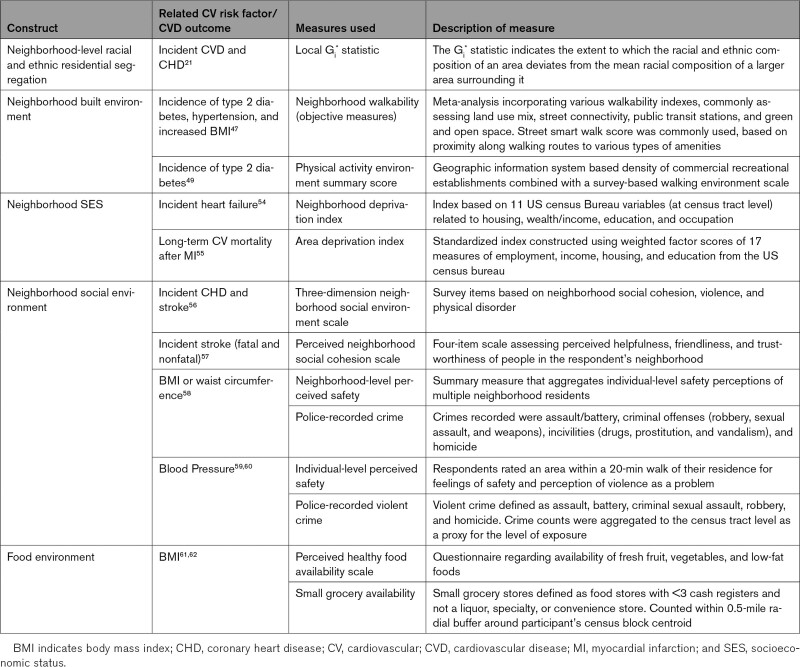
Environmental Determinants of Cardiovascular Disease: Evidence from Longitudinal Studies

**Table 2.**
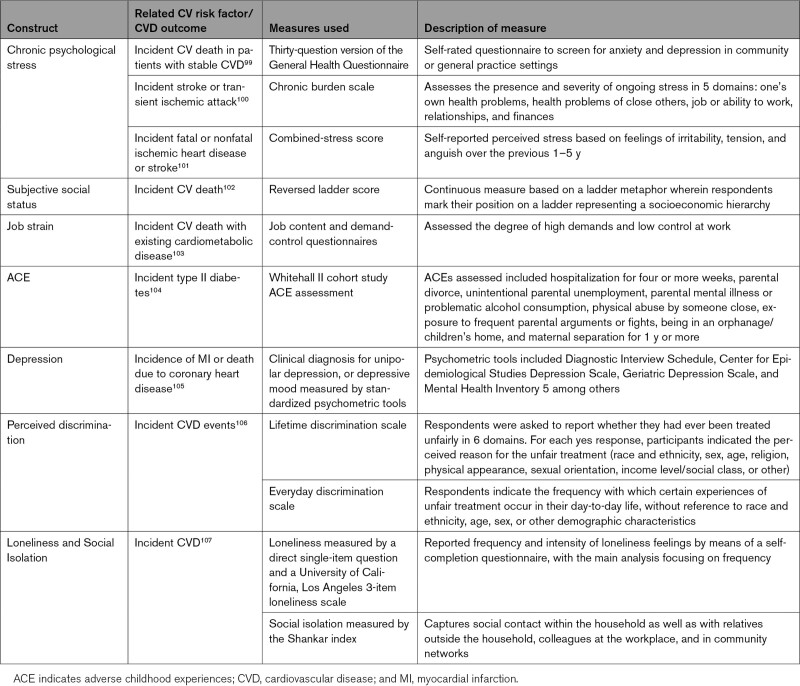
Psychosocial Factors as Social Determinants of Health: Evidence From Longitudinal Studies

**Table 3.**
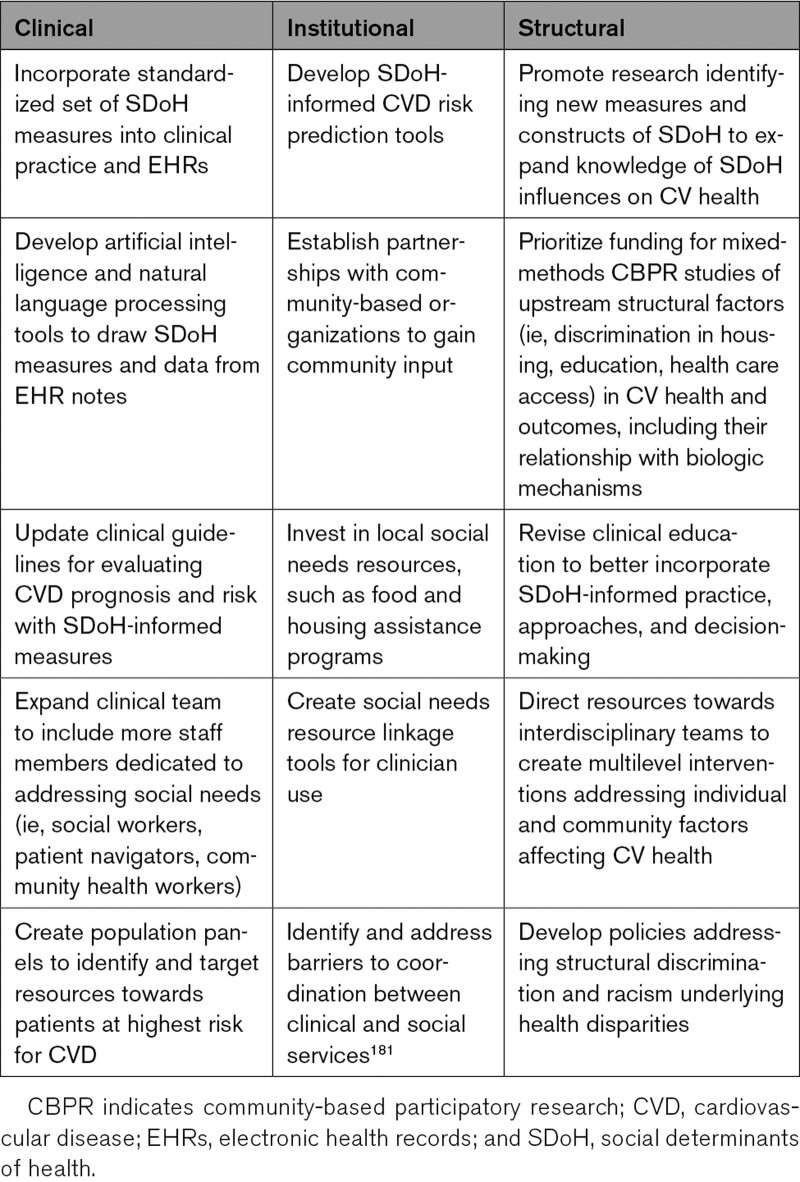
Directions for Future Research

## The Elements of an Equity-Focused Social Determinants of Health Framework

### Structural Racism and Structural Discrimination

Structural racism refers to laws, policies, and practices that were borne out of a history of discrimination and are now embedded within economic, cultural, and societal norms.^17,18^ Socioeconomic factors and, subsequently, other SDoH are influenced by structural determinants of health, or policies and systems put in place that dictate access to these resources. The links between structural racism and health inequities have been well documented.^18–20^ One way structural racism influences cardiovascular health of Black populations is through racial residential segregation.^21^ The current state of residential segregation is, in part, the result of a history of redlining, where areas of Black populations were flagged as hazardous investments by the government-established Home Owner’s Loan Corporation following the Great Depression.^18^ Discriminatory zoning, mortgage discrimination, and restrictive covenants further exacerbated the situation.^17^ Although the practice of redlining ended in the late 1960s, many neighborhoods remain segregated due to a legacy of social divestment in neighborhood infrastructure which perpetuate a disadvantage for predominantly Black neighborhoods.^18,22^ Currently, residential segregation has been shown to reduce employment opportunities and economic status, restrict access to quality education, and increase levels of neighborhood violence, crime and poverty.^20^ As such, the current state of segregation perpetuates health disparities observed in populations with low SES. In addition to SES, segregation may contribute to the development of psychosocial and environmental determinants of health as detailed in the following sections. Furthermore, it is important to consider the effects of structural racism that began before residential segregation, given that county-specific legacy of slavery associates with present-day heart disease mortality and may be a manifestation of intergenerational trauma.^23^

Overall, structural racism associates with cardiovascular health.^21,23,24^ In a multi-ethnic US cohort, Black populations living in areas with higher Black segregation had a 12% higher risk of incident CVD, independent of individual SES, CVD risk factors, and neighborhood characteristics.^21^ Conversely, White populations had a 12% lower CVD risk, but this was attenuated after adjusting for neighborhood characteristics. While the attenuation suggests neighborhood poverty may be a strong factor linking segregation to cardiovascular health disparities, the association of higher incident CVD with segregation in Black populations despite adjustment for neighborhood characteristics implies there are factors outside of neighborhood poverty also affecting cardiovascular health in segregated Black populations. Future research should consider broader domains wherein structural racism may operate. When political participation, employment, educational attainment and judicial treatment at the state level were considered as measures of structural racism, Black individuals in high-structural racism states were more likely than Black individuals in low-structural racism states to report a past-year history of myocardial infarction after controlling for age, sex, education, household income, medical insurance, and state-level poverty.^24^ The authors note that more research is needed to expand on indicators of structural racism by including factors like bank lending practices, access to education, and racial profiling.

Additionally, structural discrimination and its impact on cardiovascular health may vary by race and ethnicity.^6,20^ For Hispanics/Latinx nationally in the United States, segregation has paradoxically been shown to associate with improved self-rated health (SRH) among those who were foreign-born, although poor SRH was reported in segregated US-born Hispanics/Latinx.^25^ The findings from segregated foreign-born Hispanics/Latinx may reflect the health-promoting characteristics of Hispanic/Latinx immigrant communities such as social support and protection from discrimination. In contrast, the poorer SRH reported among segregated US-born Hispanics/Latinx supports the idea that not only segregation but also social isolation may contribute to poor SRH. In a similar study investigating segregation and self-reported health in metropolitan Black individuals, it was found that social isolation had stronger associations with poorer SRH than did segregation alone.^26^

It is also important to investigate how structural factors differentially affect the cardiovascular health of various subgroups within affected populations. In the same national study of Hispanics/Latinx, US-born Mexicans and Puerto Ricans were found to be most impacted by segregation compared with other Hispanic/Latinx groups for reasons unknown. Similar subgroup heterogeneity is found among the different American Indian and Alaska Native tribal nations. While significant cardiovascular health disparities exist among American Indian and Alaska Natives overall, there are notable variations in the prevalence of cardiovascular risk factors (eg, smoking and alcohol consumption) and CVD mortality rates across different tribal nations.^27^ More research is needed to understand the differential development of health disparities within the historical context of diverse tribal nations, especially considering American Indian and Alaska Natives have experienced structural discrimination across many geographic areas stemming from a long history of European colonization and exclusionary US policies.^28^ The importance of investigating subgroups when examining segregation or structural racism is again demonstrated in a study of Americans who self-identified as Black but were further categorized as African American or Black immigrants of immediate Caribbean descent (Afro-Caribbeans). Increased residential concentration of Afro-Caribbeans was correlated with improved SRH while increased residential concentration of African Americans was correlated with poor SRH.^29^

While we have focused on structural racism, it is also important to recognize that many other forms of structural discrimination exist that can similarly affect health, such as structural gender discrimination^30,31^ and structural discrimination of individuals with disabilities.^32^

### Socioeconomic Status

Numerous investigations spanning several decades have found strong connections between individual-level socioeconomic factors as SDoH (ie, education, income, and occupation) and CVD outcomes, with more recent data suggesting that lower SES serves a source of chronic stress that promotes a proinflammatory state and atherogenesis.^10,33–35^ This effect persists throughout the life course, as childhood low SES has been found to promote CVD events in adulthood^36^; however, more work is needed to examine the early life SES relationship with CVD independent of adult SES.^37^ It is also important to examine how SES-CVD relationships can vary by regions within countries, particularly in middle-income countries where the SES-strata at highest CVD risk may differ in urban versus rural areas.^38^ In low-income countries, available epidemiological data has indicated a relationship between education and CVD events.^36^ Additional investigation on the role of SES in CVD in developing countries is needed especially given the rising hypertension, hyperlipidemia, prediabetes/diabetes, and overweight/obesity rates in these countries.^39^ These trends are likely a result of industrialization, urbanization, and the subsequent transitions in dietary patterns, all of which can have a bidirectional relationship with SES.

Access to quality education and health care are highlighted as structural determinants of health equity in Figure 1 and are both affected by socioeconomic factors.^40,41^ Health care access as measured by insurance status is a particularly important SDoH. The expansion of health insurance access with the Affordable Care Act demonstrated that expanded insurance coverage for low-income populations was associated with greater health care utilization, including improved access to primary and subspecialty care as well as increased access to prescription drugs.^41^ However, it is less clear how greater insurance access affects CVD care quality or long-term CVD outcomes.^42,43^ Recent data suggest that the Medicaid expansion with the Affordable Care Act was associated with improvements in markers of hypertension and diabetes in low-income populations.^44^ While SES is a widely studied and important factor in both access to health care and CVD outcomes, we must also examine the intersection of this factor with other barriers like structural racism. Structural racism not only further reinforces and perpetuates the observed health disparities we see with low SES but also contributes unique damaging effects independent of SES.^19^

### Neighborhood Environment

Neighborhood built environment, defined as the physical design of neighborhoods, is a key SDoH that influences CVD disparities.^45^ Built environments offer opportunities for physical activity through the connectedness of streets, mixture of land use, large scale environmental characteristics, and transportation systems.^46^ Recent reviews have identified certain attributes of built environments that associate with CVD risks and outcomes.^47,48^ For instance, residential density, traffic safety, recreational facilities, and street connectivity were consistently associated with physical activity and body mass index, while high density traffic and residential proximity to roads (defined as closeness from home to major heavy traffic roads) were associated with incident coronary heart disease.^48^ In Table 1, we describe longitudinal studies that found associations between neighborhood walkability or physical activity environment and type II diabetes.^47,49^ Research has also demonstrated that neighborhood greenness or vegetation is protective for cardiovascular health, possibly due to promoting physical activity and social contact, decreasing stress, and mitigating pollution, noise, and heat exposure.^50,51^ Future longitudinal studies should examine which specific built environment attributes influence factors like obesity and CVD, and how physical activity may mediate these relationships.^47^ Moreover, additional data are needed on how cardiovascular health disparities and built environment factors interact with hazardous weather phenomenon linked to climate change.^52,53^

Neighborhood-level socioeconomic environment is often measured by a neighborhood deprivation index calculated from US census data on housing, income, education, and occupation information of a neighborhood. Lower neighborhood SES has been associated with incident coronary heart disease and with incident heart failure independent of individual-level SES.^54,63^ Higher area deprivation index as a marker of lower neighborhood SES was recently associated with higher cardiovascular mortality for individuals postmyocardial infarction at or before age 50 (Table 1).^55^

Perceived neighborhood social cohesion, violence, and physical disorder as markers of neighborhood social environment have also been associated with incident coronary heart disease and stroke.^56,57^ While police-reported crime and perceived safety as neighborhood social factors have been associated with changes in blood pressure or adiposity,^58–60^ less is known about the relationship between crime and perceived safety in relation to CVD outcomes.

Most built and social environment research has exclusively focused on the residential areas of individuals, investigating the relationships between environmental attributes around the home (self-reported and objectively measured) with CVD risk and outcomes.^64^ Relying on only residential environment attributes rather than an individual’s entire outside activity space (ie, geographic areas where individuals spend time throughout the day)^65^ results in a spatial mismatch, which is a key limitation of prior built and social environment studies.^66,67^ To address this limitation, researchers have begun using global positioning systems to capture the environmental attributes of individuals’ activity space.^68–70^ Future research should focus on the built environment attributes around global positioning systems-defined activity space linked to cardiovascular health outcomes in addition to static (home-centric) approaches. Future research should also consider addressing potential causal interference from selective daily mobility bias. For example, if exposure to community parks from global positioning systems data is found to be associated with decreased body mass index, the exposure itself may not be the causal factor, rather the association may also be explained by intentional visits to the parks.^71,72^

### Food Environment

A person’s food environment defined by food access, the ability to acquire food, or food security, which is assured availability of nutritionally adequate foods acquired in socially acceptable ways, influence dietary behaviors and CVD risk factors.^73^ In our equity-focused SDoH framework, we include the food environment in the social and community context in which individuals live, considering the food environment’s relationship with lived personal experiences that influences dietary behaviors (Table 1).^61,62^ SDoH frameworks that identify the food environment as a distinct social determinant allow for further insight into the relationship between food environments and collective action, policy, and social surroundings.^74^

The number of supermarkets, smaller chain food stores, fast-food establishments, and full-service restaurants as a food environment measure has been associated with dietary intake and diet quality in longitudinal studies.^62,75–78^ These associations occur in both urban and rural areas, and predominantly in neighborhoods that are lower-income or majority Black or Hispanic/Latinx.^79–84^ Social factors, including social support and social cohesion, may also influence dietary intake depending on the built environment setting.^85^ Occupation, including work hours, additionally influences food access and dietary choice.^86,87^ Food environment and dietary intake strongly associate with biologic and psychological mechanisms of health related to SDoH domains,^88^ such as inflammation,^89^ stress response,^90^ and immune response.^91^ Important areas for future research evaluating the impact of the food environment on CVD risk factors include determining the role of an individual’s experience within the food environment, the role of activity spaces, and the impact of a person’s travel routes within nonresidential food environments on dietary behavior and other cardiovascular health markers.^92–95^

### Psychosocial Factors

It is well-established that psychosocial factors, or characteristics influencing individuals psychologically or socially,^96^ are significantly associated with cardiovascular health outcomes (Table 2),^97^ both directly through chronic activation of physiological stress responses and systemic inflammation (Figure 2) and indirectly by increasing the frequency of behaviors with potentially negative effects on cardiovascular health.^98^ Longitudinal studies have demonstrated numerous psychosocial determinants of cardiovascular health, including chronic psychological stress,^99–101^ subjective social status,^102^ job strain,^103^ adverse childhood experiences,^104^ depression,^105^ perceived discrimination,^106^ and loneliness/social isolation.^107^ Moreover, psychosocial risk factors differ in terms of prevalence and chronicity among socioeconomic and racial and ethnic groups, contributing to CVD disparities.^108–110^ There are also protective psychological traits like resilience and self-efficacy which have been associated with positive effects on cardiovascular health.^111^ Similarly, motivation and executive function can indirectly influence cardiovascular health by their effect on obesity-related outcomes like weight loss, weight loss maintenance, and attainment of goals to engage in physical activity.^112^

Upstream social factors that should be considered when examining psychosocial determinants of cardiovascular health include adverse childhood experiences which have negative impacts on cardiovascular health by disrupting normal developmental processes and increasing physical and behavioral vulnerability to disease.^113^ These effects have been demonstrated to persist into adulthood, as adults who have an adverse childhood experience score of 4 or higher are 2 times as likely to develop CVD.^113^

Despite the clear importance of psychosocial determinants in CVD outcomes, a standardized measure that adequately captures multiple facets of this diverse construct is currently lacking.^114^ It is particularly important to incorporate the lived experiences of marginalized populations into existing psychosocial measures to investigate populations most impacted by disparities in cardiovascular health. When considering psychosocial measures to include in interventions or clinical care, we propose incorporating key psychosocial determinants shown to be associated with CVD risk factors in longitudinal studies, as described in Table 2. More research is needed on the integration of these measures into CVD treatment algorithms and clinical practice.

## Social Determinants of Health and the Biology of Adversity

We have reviewed constructs of our framework and their associations with cardiovascular health; however, it is important to recognize that precise biologic mechanisms linking these stressors to CVD remain largely unknown. This biology of adversity is a critical component in understanding CVD development in marginalized communities. One promising avenue of investigation examines how constructs previously described may induce psychological stress (eg, discrimination, loneliness, job strain, neighborhood violence, and food insecurity^90^) and chronically activate the sympathetic nervous system (SNS). Recent studies demonstrating how the immune system responds to chronic stress provide insights into how SDoH can lead to CVD. Uncovering how adverse SDoH may affect key signaling pathways also aids in our interpretation of clinical trial data and enhances our ability to assess the efficacy of interventions via measurement of key biomarkers of adversity described in the following sections. Here, we detail innovative studies investigating how SDoH induce stress hormones, inflammation, and other cellular processes that may contribute to CVD risk.

### Social Determinants of Health and Inflammation: Stress Hormones

SDoH can act as long-term psychosocial or environmental stressors which have the capacity to alter human biology (Figure 2). It is understood that chronic psychosocial and environmental stressors activate the SNS, including the sympatho-adrenomedullary axis and the hypothalamic-pituitary-adrenal axis. Activation of these axes ultimately increases the levels of stress-related hormones including corticotropin-releasing hormone, adrenocorticotropic hormone, cortisol, and catecholamines. Dysregulated levels of these hormones have been found in individuals experiencing low SES, depression, abuse-related posttraumatic stress disorder, discrimination, neighborhood deprivation, air pollution, or limited greenspace.^51,115–118^ This chronic activation of the SNS by way of the hypothalamic-pituitary-adrenal and sympatho-adrenomedullary axes has been linked to altered stress hormone signaling that results in increased inflammation.^119^

Glucocorticoid receptor resistance results from chronic activation of the SNS and the hypothalamic-pituitary-adrenal axis^120^ and has been linked to blunting of the anti-inflammatory response, allowing for the development and progression of CVD.^120–122^ Within the context of glucocorticoid receptor resistance, this paradoxical rise in chronic inflammation associated with increased cortisol levels may be due to immune cells becoming increasingly desensitized to cortisol through impaired nuclear translocation of the glucocorticoid receptor complex. Subsequent impairment of glucocorticoid-mediated inhibition of transcription regulated by NF-κB (nuclear factor κ-light-chain-enhancer of activated B cells) leads to increased production of proinflammatory cytokines like TNF-α (tumor necrosis factor α) or IL-6 (interleukin 6).^123^ Increased frequency of discrimination, decreased social support, social isolation, and depression have all been linked to acquired glucocorticoid receptor resistance.^119,124–126^ However, more work is needed to systematically examine the role of SDoH in the development of acquired glucocorticoid receptor resistance.

Chronic activation of the SNS also stimulates the sympatho-adrenomedullary axis which results in increased catecholamines, including dopamine, norepinephrine, and epinephrine. Catecholamines function as neurotransmitters and hormones, and are known mainly for their regulation of the fight or flight response.^127^ Norepinephrine and epinephrine signal through ARs (adrenergic receptors), a class of G-protein–coupled receptors. One subtype, the β2-AR (β2-adrenergic receptor), acts to increase intracellular cAMP which activates PKA (protein kinase A) to phosphorylate the transcription factor CREB (cAMP response element-binding protein). Under normal conditions, this cAMP-mediated process actively suppresses proinflammatory NF-κB signaling.^128^ However, evidence suggests that long-term exposure to adverse SDoH, including lower SES and social isolation, promote a switch from classical to noncanonical activation of β2-AR signaling. Noncanonical activation then redirects the β2-AR from cAMP signaling towards the G-protein-independent ERK (extracellular signal-regulated kinases) 1/2 and MAPK (mitogen-activated protein kinases) proinflammatory pathway.^119^

The relationship between SDoH and dopamine is less well studied. In both acute and chronic stress, dopamine secretion appears to vary in a corticotropin-releasing hormone-dependent manner.^129^ However, additional studies are needed to examine the impact of dopamine receptor signaling on immune cell function and subsequent CVD risk in the setting of chronic psychosocial and environmental stressors that serve as SDoH.

### Social Determinants of Health and Inflammation: Inflammatory Markers

Elevated markers of inflammation have been closely associated with both psychosocial stress^130,131^ and environmental stressors.^132^ In terms of socioeconomic determinants of health, data from the US Framingham Offspring Study cohort showed that educational level was significantly associated with CRP (C-reactive protein), sICAM-1 (soluble intercellular adhesion molecule-1), and MCP-1 (monocyte chemoattractant protein 1) in fully adjusted models.^133^ Recently, a meta-analysis found that lower SES associated with increased CRP and IL-6.^130^ In another study, exposure to low SES in early life resulted in increased CRP levels in adulthood when compared with individuals raised with higher SES.^134^

Psychosocial determinants of health like perceived stress, childhood adversity, and discrimination have also been linked to inflammatory cytokines. For instance, Casaletto et al^135^ found that high levels of perceived stress associated with various plasma cytokine levels, including IL-6, TNF-α, and MIP-1α and MIP-1β (macrophage inflammatory protein 1α and 1β).^135^ Risky emotional family environment in childhood predicted higher levels of IL-2, IL-6, IFN-γ (interferon γ), and TNF-α in adulthood^136^; childhood trauma was also found to be associated with increased IL-6 in adulthood.^137^

Discrimination specifically has been connected to inflammation in various studies.^138–140^ In a multi-ethnic study, associations between lifetime discrimination and inflammation burden were significant in models that controlled for sociodemographics, health behaviors, and psychological factors. Furthermore, poor global sleep quality was found to mediate the association between lifetime discrimination and inflammation.^141^ Future studies could examine whether disturbances in early life sleep pattern predict cardiovascular risk factors and CVD outcomes across the life course.

With regard to environmental factors and inflammatory cytokines, individuals residing in neighborhoods with higher rates of crime, violence, decreased walkability, low levels of social cohesion, decreased access to health care, increased pollutant exposure, and food insecurity display higher serum levels of CRP, IL-6, and fibrinogen.^132^ An increase in circulating IL-6 levels has especially been seen in aging adults residing in neighborhoods with racial segregation and increased poverty.^142^ In a recent study from our lab, we demonstrated that within a cohort of Black individuals, neighborhood deprivation index as a marker of neighborhood-level SES was associated with increased levels of TNF-α and IL-1β.^143^ We also determined that neighborhood deprivation was associated with trimethylamine N-oxide,^143^ a biomarker associated with the gut microbiome, cardiovascular risk factors, and CVD mortality.^144^ Interestingly, our data showed that the neighborhood deprivation index-to-trimethylamine N-oxide relationship was significantly mediated by the TNF-α and IL-1β response,^143^ suggesting a need for future work to examine how inflammation induced by adverse SDoH may relate to the gut microbiome and CVD risk. Ultimately, more work is needed to link neighborhood factors to inflammatory markers and immune cell function.

### Social Determinants of Health and Immune Cell Function

Inflammation and immune cell function are tightly connected; therefore, one would expect that SDoH impact the immune cell landscape in the human body. It is likely that SDoH could impact proliferation or clonal hematopoiesis^145^ within the bone marrow and spleen, and thus affect the distribution and function of immune cells. By using 18F-fluorodeoxyglucose positron emission tomography/computed tomography imaging, we and others have found that increased amygdalar activity, a marker of chronic stress-related neural activity, associates not only with subclinical CVD but also with heightened bone marrow and splenic activity.^146,147^ This highlights the importance of the neuro-hematopoietic-inflammatory axis in CVD development and progression (Figure 2). One possible reason for the increased metabolic activity detected in the bone marrow of those with high chronic stress is stem cell proliferation and clonal hematopoiesis, which have been associated with inflammation and CVD in mouse models of atherosclerosis.^145^ Moreover, cytokines likely play a role, given that lower IL-6 in humans has been associated with decreased clonal hematopoiesis and subsequent decreases in CVD risk.^148^ More work is needed to investigate any potential impact of SDoH on the distribution of immune cell populations and sub-types as well as immune cell receptor expression associated with CVD.

### Social Determinants of Health and Acceleration of Cellular Aging

It is also critical to understand how DNA or epigenetic modifications might emerge due to a lifetime of disadvantage, structural inequalities, and discrimination. Biologic effects of SDoH on CVD risk may be passed on intergenerationally or can be related to gene-environment interactions.^149^ Moreover, the interaction between inflammation, age, and clonal hematopoiesis drives the hypothesis that SDoH and the exposure to chronic psychosocial and environmental stressors may accelerate cellular aging. The link between inflammation, aging, and CVD was further established by Sayed et al^150^ in their inflammatory aging clock, which is characterized by several markers of aging including epigenetic modifications and shortening telomere length. Prior studies have tied epigenetic aging of monocytes, marked by DNA methylation, to low SES in early life.^151,152^ Furthermore, using data from the MESA study (Multi-Ethnic Study of Atherosclerosis), Schmitz et al^153^ determined that epigenetic aging is accelerated in individuals experiencing socioeconomic disadvantage. Similar associations with accelerated epigenetic aging can be found at neighborhood-level SES.^154^ Investigations that describe DNA methylation patterns among individuals living in disadvantaged neighborhoods have shown alteration of gene expression particularly among genes involved in inflammatory pathways.^155^

In addition to DNA methylation, another cellular aging marker used to determine the impact of SDoH on genes is telomere length,^156^ which has been linked to various chronic diseases, including CVD.^157^ Using self-identified race,^158^ Black individuals display greater telomere length at birth when compared with White individuals; however, this difference diminishes over the lifetime due to an increased rate of telomere shortening among Black individuals.^159^ Inflammation and oxidative stress, which are promoted by chronic psychosocial and environmental stress exposure, are likely the main mechanisms that promote loss of telomere length.^160–162^ Thus, numerous psychosocial and environmental stressors have been connected to telomere length or telomerase activity, including neighborhood deprivation,^154,163^ neighborhood disadvantage,^164^ low SES and educational level,^165,166^ early life stress,^167^ lower social support,^168^ increased early life adversity,^169^ high hostility,^170^ anxiety,^171^ and racial discrimination.^168,172^ Moreover, the association between lower parental SES and shortened telomere length in the newborn^166^ further demonstrates that the biologic effects of SDoH can reach across generations and influence health even before direct exposure to stressors. Future work should focus on the mechanisms by which SDoH affect telomere length so that targeted interventions may not only prevent development and progression of telomere shortening but also block this intergenerational transmission of adversity.

Overall, it is important for us, as clinicians and scientists, to address the relationship between SDoH and cardiovascular health at multiple levels of intervention. On a biologic level, translational studies should include SDoH measures, and intervention studies would benefit from investigating effects on key biomarkers of stress and adversity. Animal studies are also important to study the biology of adversity in a more controlled environment. Furthermore, we must complement biologic investigations with targeted population health interventions addressing SDoH constructs that are known contributors to biologic adversity.

## The Role of Interventions in Addressing the Impact of Social Determinants of Health

Although it is well-established that incorporating SDoH screening and interventions into chronic disease clinical care significantly improves patient outcomes, current guidelines largely exclude SDoH-informed approaches.^173^ The National Academies of Sciences, Engineering, and Medicine proposed a framework for improving social care through awareness of patients’ social needs through screening, adjustment of care to patients’ individual contexts, connecting patients to community resources, enabling institutions to prioritize resources in line with patient needs, and promoting policies that expand social care resources.^174^ Additionally, the American Heart Association has suggested expanding SDoH education for cardiovascular health providers at all levels, improving tools using electronic health records to incorporate SDoH screening and referrals into clinical practice, and expanding SDoH interventions to address upstream determinants of CVD such as poverty, education, and health care coverage.^175^

Previous studies demonstrated the success of efforts such as community health worker or patient navigator programs,^176^ social risk score assessments,^177,178^ and health behavior counseling^179^ on reducing blood pressure, CVD risk, and LDL (low-density lipoprotein) levels.^180^ For example, the RICH LIFE (Reducing Inequities in Care of Hypertension: Lifestyle Improvement for Everyone)^176^ intervention sought to reduce hypertension control disparities through a collaborative care model that involved nurse care managers to coordinate care for patients with comorbidities, whereas the WISEWOMAN (Well-Integrated Screening and Evaluation for Women Across the Nation)^179^ intervention introduced lifestyle counseling from bilingual community health workers to improve cardiovascular behaviors among Latina women. Other interventions have targeted systematic approaches to SDoH integration in clinical care, such as developing a modified version of the Framingham CVD Risk Score^177^ and adding measures of SDoH to improve the predictive accuracy of CVD risk models.^178^ Major opportunities for integrating SDoH into clinical cardiovascular care can be found in standardizing electronic health records-based tools for SDoH assessments, facilitating panel management to identify and direct outreach to high risk patients, and tailoring clinical decisions to address environmental factors like housing conditions and health literacy.^181,182^ Clinical guidelines for CVD management can be updated to incorporate SDoH-informed care practices. Moreover, clinical education can be revised to better inform health care providers about how to identify and address their patients’ social needs. Health care institutions can also strengthen partnerships with community-based organizations to support the availability of social needs resources.

Following a community-based participatory research approach,^183^ community input about needs and priorities should play a major role in the process of developing informed interventions at the individual, community, and policy levels. Ultimately, these interventions should contribute to multilevel strategies addressing cardiovascular health based on the foundation of community consensus and partnership.^184^ Consultation of community members can also provide insight into protective factors that critically influence health behaviors and outcomes,^185^ including community capacity and empowerment.^186^

Mixed methods approaches combining qualitative and quantitative data are also critical in cardiovascular intervention development that incorporate SDoH. Over a decade ago, the Office of Behavioral and Social Sciences Research of the National Institutes of Health commissioned leaders in the mixed methods field to develop guidance for investigators on the rigorous elements necessary to conduct mixed methods research. Mixed methods approaches included (1) focusing on research questions that call for real-life contextual understandings, (2) using and integrating both rigorous quantitative and qualitative methods to fully understand and characterize constructs, and (3) framing the investigation within philosophical and theoretical positions.^187^

In SDoH research, it is important to recognize that focusing only on quantitative variables provides neither a holistic nor accurate portrait of the person, family, or community. Understanding biologic mechanisms of CVD is necessary but not sufficient in designing health behavior interventions and public health policy to address CVD and SDoH. There are factors in the Critical Framework of Social Determinants of Health (Figure 1) and thus in the Social Determinants of Health and the Biology of Adversity (Figure 2) that can only be discovered through a mixed methods approach. We must deliberately integrate and combine quantitative and qualitative inquiry to understand both direct and indirect contributions of SDoH to chronic inflammation pathways and CVD. Figure 3 outlines the possibilities for SDoH-informed CVD interventions at the policy, community, and individual levels. Table 3 identifies directions for future SDoH-informed research in CVD at the levels of individual clinicians and clinics, health care institutions, and research infrastructure. Ultimately, success of these intersectional practices relies on addressing major barriers to intervention implementation in vulnerable communities which are availability, accessibility, and affordability of resources for both practitioners and patients.^188^

## Conclusions

SDoH have a significant impact on CVD risk and outcomes, particularly among marginalized communities. To address health disparities and promote health equity, it is essential to understand the various facets of SDoH, including the structural health determinants, built, food, and social environments, as well as the socioeconomic and psychosocial determinants of health. In addition to improving the measurement of SDoH in cardiovascular research and care, there is opportunity to build interdisciplinary teams that further investigate relationships between SDoH and the biologic mechanisms by which these determinants affect CVD risk and outcomes. Moreover, SDoH screening should be integrated into clinical care delivery, encouraging clinicians to tailor care delivery to the social needs of their patients. Finally, future research on the SDoH-CVD relationships should incorporate mixed-method approaches to better understand how individual-lived experiences of marginalization and discrimination affect cardiovascular health outcomes and develop tailored interventions informed by a nuanced understanding of social and environmental influences on cardiovascular health.

## Article Information

### Acknowledgments

We acknowledge our partners in the Washington DC Cardiovascular Health and Obesity Collaborative community advisory board, study participants, and former students and fellows, without whom none of our work would have been possible. We also like to thank the National Institutes of Health (NIH) staff and clinical teams and our collaborators for their continuous support.

### Sources of Funding

The statements and contents expressed in this perspective are those of the authors and do not reflect the official position of the National Institutes of Health (NIH), DHHS, and the US Government. The Social Determinants of Obesity and Cardiovascular Risk Laboratory is funded by the Division of Intramural Research of the National Heart, Lung, and Blood Institute and Intramural Research Program of the National Institute on Minority Health and Health Disparities. The Translational, Biobehavioral and Health Disparities Branch is funded by the Intramural Research Program of the National Institutes of Health, Clinical Center. The Neighborhood Social and Geospatial Determinants of Health Disparities Laboratory is supported by the Intramural Research Program, National Institute on Minority Health and Health Disparities, National Institutes of Health and by the NIH Distinguished Scholars Program. The Translational Biobehavioral and Health Disparities Branch is supported by the National Institutes of Health, Clinical Center. This research was made possible through the NIH Medical Research Scholars Program, a public-private partnership supported jointly by the NIH and generous contributions to the Foundation for the NIH from the Doris Duke Charitable Foundation, Genentech, the American Association for Dental Research, the Colgate-Palmolive Company, Elsevier, alumni of student research programs, and other individual supporters via contributions to the Foundation for the National Institutes of Health.

### Disclosures

None.
